# Supplementary material for: A Systematic Review of the Effect of Therapeutic Drug Monitoring on Patient Health Outcomes during Treatment with Carbapenems
Source: Antibiotics (Basel). 2022 Sep 27;11(10):1311. doi: 10.3390/antibiotics11101311 (PMC9598625; doi:10.3390/antibiotics11101311)
Supplement: Supplementary file 1 [file antibiotics-11-01311-s001.zip › antibiotics-1931260-supplementary.pdf]

Supplementary Materials

Supplementary Material S1 – Database Search

Ovid MEDLINE(R) ALL 1946 to February 7, 2022

Searched: 07Feb22

1 - exp Carbapenems/ (13305)

2 - (meropenem* or imipenem* or ertapenem* or doripenem*).tw. (17863)

3 – 1 or 2 (23614)

4 - exp Drug Monitoring/ (22867)

5 - (TDM or drug monitoring).tw. (12330)

6 - (dos* adjustment* or dos* alteration* or dos* escalation*).tw. (23759)

7 - (personal* adj2 (medicine or therap* or treatment* or dos*)).tw. (32519)

8 - (custom* adj2 (medicine or therap* or treatment* or dos*)).tw. (1888)

9 - (individual* adj2 (medicine or therap* or treatment* or dos*)).tw. (45062)

10 - 4 or 5 or 6 or 7 or 8 or 9 (128383)

11 - 3 and 10 (372)

12 - limit 11 to yr="2020 -Current" (109)

Embase Classic+Embase 1947 to 2022 February 7

Searched: 07Feb22

1 - exp carbapenem derivative/ or exp carbapenem/ (23792)

2 - (meropenem* or imipenem* or ertapenem* or doripenem*).tw. (27501)

3 - 1 or 2 (46212)

4 - exp drug monitoring/ (57056)

5 - (TDM or drug monitoring).tw. (19008)

6 - (dos* adjustment* or dos* alteration* or dos* escalation*).tw. (49267)

7 - (personal* adj2 (medicine or therap* or treatment* or dos*)).tw. (49440)

8 - (custom* adj2 (medicine or therap* or treatment* or dos*)).tw. (2972)

9 - (individual* adj2 (medicine or therap* or treatment* or dos*)).tw. (69053)

10 - 4 or 5 or 6 or 7 or 8 or 9 (225295)

11 - 3 and 10 (891)

12 - exp animals/ not exp human/ (5688639)

13 - exp nonhuman/ not exp human/ (4924602)

14 - exp experimental animal/ (778216)

15 - exp veterinary medicine/ (61459)

16 - animal experiment/ (2775796)

17 – or/12-16 (8179141)

18 - 11 not 17 (846)

19 - limit 18 to yr="2020 -Current" (182)

Cochrane

Search Name:
Date Run: 07/02/2022 16:09:26
Comment:
ID Search Hits
#1 MeSH descriptor: [Carbapenems] explode all trees (740)
#2 (meropenem* or imipenem* or ertapenem* or doripenem*).tw. (10104)
#3 #1 or #2 (10841)
#4 MeSH descriptor: [Drug Monitoring] explode all trees (1899)
#5 ("drug monitoring" or TDM):kw,ti,ab (3477)
#6 ("dose adjustment*" or "dose escalation*" or "dose alteration*"):kw,ti,ab (10304)
#7 ((personal* or custom* or individuali?ed*) NEAR/2 (medicine or therap* or treatment* or dos*)):ti,ab,kw (5616)
#8 #4 or #5 or #6 or #7 (19108)
#9 #3 and #8 (129)
#10 #9 (with publication year from 2020-2022, in Trials) (14)

Web of Science

Web of Science 07Feb22
CORE collection

A simultaneous search of the following databases:

- Science Citation Index Expanded (SCI-EXPANDED) --1900-present
- Social Sciences Citation Index (SSCI) --1900-present
- Arts & Humanities Citation Index (A&HCI) --1975-present
- Conference Proceedings Citation Index- Science (CPCI-S) --1990-present
- Conference Proceedings Citation Index- Social Science & Humanities (CPCI-SSH) --1990-present
- Emerging Sources Citation Index (ESCI) --2015-present

1 TS=(carbapenem?) (7005)
2 TS=(meropenem*) or TS=(imipenem*) or TS=(ertapenem*) or TS=(doripenem*) (18503)
3 #1 or #2 (22751)
4 TS=("drug monitoring") or TS=(TDM) (20286)
5 TS=("dos* adjustment*") or TS=("dos* escalation*") or TS=("dos* alteration") (29337)
6 TS=(personal* NEAR/2 (medicine or Therap* or treatment* or dos*)) (46060)
7 TS=(custom* NEAR/2 (medicine or Therap* or treatment* or dos*)) (2782)
8 TS=(individual* NEAR/2 (medicine or Therap* or treatment* or dos*)) (65369)
9 #4 or #5 or #6 or #7 or #8 (157075)
10 #3 and #9 (443)
#11 #3 and #9 (publication date between 2020 and 2022) (148)

Table S1 – Study characteristics of randomised controlled trials (RCTs) assessing the role of therapeutic drug monitoring during therapy with carbapenem class antibiotics.

Reference	Population	Carbapenem	TDM measurement	Concomitant Antibiotics	Renal Function	Pathogen and MIC	Pharmacological Target	Pharmacological Target Attainment	Microbiological Resolution	Clinical Resolution	Emergence of Antimicrobial Resistance
Fournier *et al,* 2018. [39]<br><br>RCT evaluating the impact of therapy guided by TDM has on target attainment in burns patients, clinical outcomes were also reported. **	Burns patients receiving IV antibiotics for a range of infections, mainly pneumonia (24) and skin/soft tissue infection (7) (n=38)<br><br>Patients receiving carbapenems n=17<br><br>Intervention group median SAPS II score: 31.0 (IQR: 22.0-42.0).<br><br>Control group median SAPS II score: 28.0 (IQR: 20.0-45.0)	Ertapenem, n=2, empiric dose: 1g q24h<br><br>Imipenem-cilastatin, n=2, 500mg-1000mg q6h<br><br>Meropenem, n=16, 1-2g q8h	Total serum antibiotic concentrations measured every other day using HPLC-MS/MS. Unbound concentrations calculated using published protein binding data.<br><br>Samples taken every two days for TDM measurement.	Not specified	Not specified	Most commonly isolated pathogens: *S. pneumoniae*, *S. aureus, Pseudomonas aeruginosa* and *Enterobacteriales*	Predefined pharmacological trough target set by antibiotic:<br>- Ertapenem:<br>Min level: 5 mg/L<br>Max level: Unknown<br>- Imipenem-cilastatin:<br>Min level: 2 mg/L<br>Max level: Unknown (8 mg/L)<br>- Meropenem:<br>Min level: 2 mg/L<br>Max level: Unknown (8 mg/L)<br><br>Intervention group dose adjusted by increasing or decreasing dose amount or frequency, or by adjusting infusion duration.<br>In the standard-care group clinicians could request a rescue TDM result in special cases.	Of initial TDM results in the intervention group 5 (29.4%) were outside the target range, 1 (5.9%) too low and 4 (23.5%) too high. After intervention, in subsequent TDM results, 7 were outside the target range (17.5%), with 3 (7.5%) too low, and 4 (10%) too high.<br><br>Of initial TDM results in the standard care group 8 (50%) were outside the target range, 6 (37.5%) too low, and 2 (12.5%) were too high. Of subsequent TDM results, where the clinicians were blinded to results, 15 (46.9%) were outside the target range, with 12 (37.5%) too low and 3 (9.4%) too high.	Not specified	No difference seen in clinical outcomes between the two groups. 33/36 (91.7%) successfully treated infection episodes in the intervention group, 30/31 (96.8%) infection episodes in standard-of-care group. Includes infection episodes from non-carbapenem antibiotics.<br><br>Infection episode cures from meropenem use in the TDM group were 14/15, versus 13/14 in the standard group. From imipenem use there were 1/1 cures to episodes in both groups. From ertapenem use there were 1/1 cures in the intervention group and 1/2 cures in the	Not specified

										standard care group.	
De Waele *et al*, 2014 [40]<br><br>RCT evaluating the impact of altering dosing regimens according to TDM in non-renally impaired patients receiving piperacillin and meropenem. **	Non-renally impaired patients receiving meropenem (and piperacillin) n=41<br><br>Intervention group median APACHE II score: 19 (IQR:12-24)<br><br>Control group median APACHE II score: 17 (IQR: 13-23) [p=0.557]	Meropenem, n=13 Extended infusion 1g infused over 3 hours every 8 hours	Daily total antibiotic concentrations determined through HPLC-MS/MS.<br><br>Unbound antibiotic concentrations not determined, total amounts used.<br><br>Samples taken daily. First sample taken after ≥3 completed infusions to reach steady-state. Samples taken mid-dosing interval and trough during the first 3 days of dosing. During final 4 days of dosing only trough samples taken.	Not specified	Renally impaired patients (eGFR <80mL/min) excluded from the study.<br><br>Intervention group day 1 median creatinine clearance: 130 mL/min (IQR: 92-177 mL/min), day 3 median creatinine clearance: 155 mL/min (IQR: 83-182 mL/min).<br><br>Standard care group day 1 median creatinine clearance: 108 mL/min (IQR: 88-145 mL/min) [p=0.291], day 3 median creatinine clearance: 110 mL/min (IQR: 90-165 mL/min) [p=0.697].	The most commonly isolated bacterial pathogens: *E. coli* (n=7), *Klebsiella pneumoniae* (n=7), *Pseudomonas aeruginosa* (n=6). Total of 43 causative pathogens isolated from 27 patients. Median meropenem MIC: 0.125 mg/L (IQR: 0.125 – 0.690 mg/L).	Pharmacokinetic targets: 100% *ƒ*T>4-10xMIC and 100% *ƒ*T>MIC, both used in the analysis.<br><br>If TDM result was <4xMIC then meropenem dosing frequency was increased, followed by an increase of dose by 50%, if subsequent TDM result was too low.<br><br>If TDM result was >10xMIC dosing frequency was decreased, if subsequent TDM result was still above 10xMIC then the dose was reduced.	Initially, in the intervention group there was a target attainment of 9.5% and 68% for 100% *ƒ*T>4xMIC and 100% *ƒ*T>MIC targets, respectively. The control group had a target attainment of 20% and 70% for targets 100% *ƒ*T>4xMIC and 100% *ƒ*T>MIC, respectively. After 72 hours, in the intervention group there was a target attainment of 58% versus 16% in the standard care group (p=0.007) for 100% *ƒ*T>4xMIC. 95% of patients met the 100% *ƒ*T>MIC target in the intervention group compared to 68% in the control group (p=0.045).<br><br>TDM significantly improved PK target attainment. **	In the intervention group there was bacterial persistence at day 7 in 1 patient. In the control group there was bacterial persistence in 5 patients (p=0.09). **	In the intervention group clinical failure was seen in 2 patients, compared to 4 in the control group (p=0.41). Median SOFA scores in the intervention group changed from 5.5 at the baseline to 3 at day 7 (p=0.093). In the control group the median SOFA score change was from 5 to 4. from baseline to day 7 (p=0.575). In the intervention group 4.8% (n=1) of patients died in the ICU compared to 20% (n=4) in the control group (p=0.18). In the intervention group 3 patients died within 28-days compared to 5 deaths in the control group (14.3% versus 25%, p=0.45).**	Not specified

Abbreviations: APACHE II, Acute Physiology and Chronic Health Evaluation II; eGFR, estimated Glomerular Filtration Rate; HPLC-MS/MS, High-Performance Liquid Chromatography coupled Tandem Mass Spectrometry; IV, Intravenous; MIC, Minimum Inhibitory Concentration; PK, Pharmacokinetic; SAPS II, Simplified Acute Physiology Score; SOFA, Sequential Organ Failure Assessment; TDM, Therapeutic Drug Monitoring; **, study included non-carbapenem antibiotics in analysis.

Table S2 – Study characteristics of non-randomised studies where TDM has been used to guide carbapenem therapy

Reference	Population	Carbapenem (number of patients) and dosing regimens	TDM sampling/ measurement	Concomitant Antibiotics	Renal Function	Pathogen and MIC	Pharmacological Target	Pharmacological Target Attainment	Microbiological Resolution	Clinical Resolution	Emergence of Antimicrobial Resistance
Cies *et al*, 2018. [43]<br><br>Observational study investigating dosing regimen effectiveness and the effect of TDM on clinical outcomes.<br><br>Analysis divided patients into two cohorts: 1) the total cohort, comprising all included patients, and 2) the infected cohort, those where a pathogen was isolated.<br><br>**, n=65	Paediatric critically ill patients and PICU patients receiving extracorporeal membrane oxygenation or CRRT, n=17<br><br>Total cohort: Median Pediatric RISk of Mortality score: 17 (range: 2-39)<br><br>Confirmed infection cohort: Median Pediatric RISk of Mortality score: 21 (range: 5-32)	Doripenem, (n=1)<br><br>Meropenem, (n=16)<br><br>Standard dosing not specified.<br><br>Intermittent dosing: 30-60 min infusion.<br><br>Extended infusion: 3-4hr infusion.<br><br>Continuous infusion: 24 hr infusions	Total plasma antibiotic concentrations determined by LC-MS/MS, free fraction was estimated using published protein binding data.<br><br>≥2 samples obtained per patient.<br><br>Intermittent dosing:<br>First samples taken 20-120 mins of end of infusion and ≥1 hr after first sample.<br><br>Extended infusion (3-4hr infusion): First samples were taken at the end of infusion, and ≥1 hr after first sample.<br><br>Continuous infusion:<br>Samples taken ≥8 hrs.	Concomitant antibiotics: vancomycin, gentamicin, and tobramycin, administered.<br><br>No analysis of effect of concomitant antibiotics on outcome, nor any reporting of concomitant antibiotic administration between cohorts.	Not specified.	Confirmed pathogens: 23/82 patients. Pathogens treated with meropenem (meropenem MICs): *Pseudomonas aeruginosa* (n=3, MIC: 0.25 mg/L, 0.25 mg/L, 8 mg/L); *Escherichia coli* (n=1, MIC: 0.24 mg/L); *Acinetobacter* spp. (n=1, MIC: 1 mg/L); *Klebsiella pneumoniae* (n=1, MIC: 2 mg/L). *Serratia* spp.(n=1, MIC: 0.25 mg/L).<br><br>Where an organism was not isolated, worst case CLSI breakpoint value was used, value not reported.	40% ƒT>4-6xMIC<br><br>Dosing frequency was reduced if meropenem concentration was too high and increased if too low, or length of infusion extended if too low.	Across the total cohort 95% (78/82) of treatments in patients failed to reach the 40% fT>4-6xMIC target.<br><br>Of the 78 patients with treatments outside the target range 5 (6.4%) had a concentration >6xMIC, and 73 (93.6%) had a concentration <4xMIC.<br><br>No patients with a pathogen identified met the pharmacological target.<br><br>Changes in target attainment were not reported following dose adjustments.	In patients where a pathogen was isolated, there was a positive microbiological response in 100% of cases.	In patients where a pathogen was isolated, there was a positive clinical response of 95.7% (22/23), resulting in a mortality rate of 4.3%. The mortality rate of the total cohort was 12.2%.<br><br>Of the whole cohort, including non-carbapenem antibiotics, no statistically significant association between target attainment and clinical response or mortality was reported.	Not specified

Machado *et al* 2017 [44]<br><br>A retrospective before-and-after study looking at clinical differences between patients before and after beta-lactam TDM was introduced.<br><br>**, n=76	Burns patients, n=140<br><br>Conventional treatment group median APACHE II score: 18 (range: 8-36)<br><br>TDM group median APACHE II score: 20 (5-26) [p=0.63]	Imipenem (n=33)<br><br>Meropenem (n=2)<br><br>Standard dosing not specified.	Plasma antibiotic concentrations determined by HPLC.<br><br>After a minimal 5 half-lives, six serial samples were taken during the course of the dosing interval.<br><br>Not specified whether or not serial antibiotic sampling was carried out more than on one occasion.	Not specified	Not specified	TDM intervention group (n=77) : *Acinetobacter baumannii* (n=12, 39%), *Staphylococcus aureus* (n=5, 16%), *Pseudomonas aeruginosa* (n=2, 6%), *Enterobacteriaceae* (n=8, 26%), other (n=4, 13%). Conventional treatment group (n=63): *Acinetobacter baumannii* (n=7, 25%), *Staphylococcus aureus* (n=9, 33%), *Pseudomonas aeruginosa* (n=4, 14%), *Enterobacteriaceae* (n=5, 18%), other (n=2, 10%)<br><br>A number of hypothetical MIC concentrations, based on clinical breakpoints, were used to determine target attainment for different susceptibilities	A pharmacological target of 60% ƒT>MIC was used for imipenem and meropenem.<br><br>Dose adjustment protocol not specified.	No target attainment data for the conventionally treated group as TDM was not carried out.<br><br>Likely target attainment modelled on a range of hypothetical MICs. For imipenem 88% would have achieved target attainment if MIC was 4 mg/L, 100% would have achieved the target with a hypothetical MIC of ≤2 mg/L.<br><br>For meropenem 90% would have achieved target attainment with a hypothetical MIC of 4 mg/L, 100% would have achieved the target with ≤2 mg/L.<br><br>No data reported on target attainment before and after dose adjustment, of those where dosing required adjustment.	Not specified	There were no significant differences in clinical outcome between the conventionally treated group and the TDM intervention group.<br><br>Conventional treatment group: hospital mortality 36%, TDM group: hospital mortality 39%, p=0.83. Conventional treatment group 14-day mortality: 14%, TDM group: 14-day mortality 16%, p=0.99.<br><br>Conventional treatment group clinical improvement: 52%, TDM group clinical improvement: 60%, p=0.37.	Not specified
Economou *et al* 2017 [45]<br><br>Study describing	Patients undergoing continuous renal replacement therapy	Meropenem, 0.5-1g q6h-q12h	Total and unbound serum concentrations of antibiotic	Concomitant antibiotics not specified.	Mean serum creatinine: 205.2 μmol/L (range: 36-711 μmol/L).	Most commonly identified pathogens: MSSA (n=11), *Klebsiella pneumoniae*	100% fT>MIC – 100% fT≤10xMIC<br><br>When measured antibiotic concentration was	Dose adjustment required in 35% of patients, 24% of adjustments needing a decrease **	Not specified	8 patients receiving meropenem died, for these patients the pathogens had a	Not specified

how dosing regimens guided by TDM effects patients receiving CRRT.<br><br>**	(CRRT), n=76<br><br>Patient condition not specified.		measured by HPLC.<br><br>Trough sample taken after 4 doses, at steady-state.<br><br>Frequency of sampling measurement was not specified.		CVVHDF settings:<br>Mean +/- SD Q_b (mL/min): 192 +/- 22 (range: 150-250)<br>Mean +/- SD Q_d (mL/min): 346 +/- 524 (range: 1000-3000)<br>Mean +/-SD Q_f (mL/min): 144 +/- 110 (range: 0-1000)<br>Mean +/- SD Post-dilution rate (mL/h): 1532 +/- 739 (range: 50-3375)<br>Mean +/- SD Pre-dilution rate (mL/h): 1222 +/- 441 (range: 1000-2000).<br><br>No analysis of effects of CVVHDF on target attainment was carried out.	(n=6), *Escherichia coli* (n=14), *Pseudomonas aeruginosa* (n=3), *Enterococcus faecalis* (n=4).<br><br>73% (n=81) of microorganisms were identified, 27% (n=30) were not identified.<br><br>Where possible MIC values determined through VITEK2, otherwise EUCAST susceptibility breakpoints were used. The highest susceptible MIC was used from potential pathogens was used when no microorganism was isolated.	outside of desired range, doses were accordingly increased or decreased. Specific dose adjustment protocol not specified.	In meropenem patients TDM results, 25 dosing regimens (65.8%) were maintained, 6 (15.8%) were increased and 7 (18.4%) were decreased.<br><br>Not reported whether dose changes improved target attainment.		median MIC of 2 mg/L (IQR: 2-2 mg/L). Median unbound trough concentrations for patients that died (fC_{min}): 6x MIC (IQR: 4.5x MIC – 8.5xMIC).<br><br>No statistical associations were made between achieved adequate concentrations and clinical outcomes.	
Fournier, A. *et al*, 2015. [46]<br><br>Study looking at the impact of TDM in critically ill burn patients treated with	Critically ill burn patients n=109, (TDM: n=27, standard care: n=82).<br><br>TDM patients were more severely burned (median	Imipenem-cilastatin (n=48, TDM patients n=4, standard care patients n=44).<br>Empiric dose: 500mg q6h, 30 min infusion.	Before 2010 antibiotic plasma concentrations determined by HPLC-UV, available once per week, after 2010 UPLC-MS/MS used and testing	Concomitant antibiotics not specified	Median creatinine level in imipenem infection episodes with at least one antibiotic concentration measurement: 46 µmol/L	Common pathogens isolated from imipenem-cilastatin infection episodes with at least one antibiotic concentration measurement:	Dosages adjusted to exceed MIC levels. If there were no determined MIC trough serum concentrations were adjusted to >1 mg/L prior to 2009, from 2009 onwards this was increased to >2	Of imipenem-cilastatin infection episodes with at least one antibiotic concentration measurement (n=13), 7/13 had appropriate levels. Of those with inappropriate levels, 6/6 were lower than targets,	Not specified.	Of imipenem-cilastatin infection episodes with at least one antibiotic concentration measurement , 11/13 resulted in clinical success, 2/13 resulted in	Not specified.

carbapenem antibiotics.	TBSA: 47%) than standard care (median TBSA: 25%), p<0.0001.<br><br>TDM patients: mean Ryan score of 1.41 +/-0.69, standard care patients: 0.95 +/- 0.63.	Meropenem (n=38, TDM patients n=12, standard care patients n=26). Empiric dose: 1g q8h, 30 min infusion.<br><br>Imipenem-cilastatin and meropenem (n=23, TDM patients n=11, standard care patients n=12).	available 4 days a week.		(IQR:33.5 - 63.5 µmol/L).<br><br>Median creatinine level in meropenem infection episodes with at least one antibiotic concentration measurement: 72 µmol/L (IQR:52 - 156 µmol/L).<br><br>Renal function of standard care patients not specified.	*P. aeruginosa*, (n=6).<br><br>Common pathogens isolated from meropenem infection episodes with at least one antibiotic concentration measurement *P. aeruginosa*, (n=15); *E. cloacae* (n=4).<br><br>Pathogens isolated from standard care patients not specified.	mg/L, in accordance to the EUCAST MIC_{90} of local commonly found pathogens.	5/6 had dosages increased, in 1/6 treatment was de-escalated. Subsequent TDM measurements showed that trough levels fluctuated from appropriate to inappropriate despite dose increases.<br><br>Of meropenem infection episodes with at least one antibiotic concentration measurement (n=23), 7 cases showed levels that were too high and 7 cases showed insufficient levels. There was variable success in achieving target levels in those with subsequent TDM measurements.		a persistent infection.<br><br>Of meropenem infection episodes with at least one antibiotic concentration measurement, 15/23 resulted in clinical success, 6/23 resulted in a persistent infection, and 2/23 resulted in failure.<br><br>Length of stay (LoS) was significantly different between groups. TDM group median LoS: 53 days (IQR: 35-78 days); standard care: 27.5 days (IQR: 16-47.25), p<0.0001.<br><br>Burn ICU mortality was not significantly different, TDM patients: 18.5% (n=5), standard care 8.5% (n=7), p=0.167.<br><br>There was no associations made between target attainment and infection outcome.	

McDonald *et al*, 2016. [47]<br><br>Retrospective study looking at TDM guided beta-lactam dosing in critically ill patients and whether high-dose therapy results in toxicity.<br><br>**, n=46	Critically ill patients, n=93<br><br>Licensed-dose group: Mean SOFA score: 6.3 (SD: +/- 3.6)<br>High-dose group: Mean SOFA score: 5.0 (SD: +/- 3.6) [p=0.20]	Meropenem, n=47<br><br>Patients categorised into licensed dose (≤3g/24h for systemic infection and ≤6g/24h for CNS infection) and high dose groups (>3g/24h for systemic infections and >6g/24 for CNS infection)	Unbound plasma concentrations determined by HPLC.<br><br>Sampling was initiated 4 half-lives after start of therapy. Frequency of TDM was not reported.	Concomitant antibiotics not specified.	Meropenem patients that received a licensed-dose CrCl: 128.4 mL/min +/- 19.9 mL/min<br><br>Meropenem patients receiving a high dose: CrCl: 234.2 mL/min +/- 94.6 mL/min [p<0.001].<br><br>Analysis of effects of renal function on target attainment not performed.	Most commonly identified pathogens in meropenem patients: *Escherichia coli* (n=8), *Klebsiella pneumoniae* (n=10), *Enterobacter cloacae* (n=6) Susceptibility data not specified.<br><br>For patients without an isolated pathogen, target MIC values were based on clinical breakpoint data of likely pathogens. Surrogate MIC value not specified.	100% ƒT>MIC<br><br>Where antibiotic concentrations were low, dosing frequency was increased by 25-50%. If the concentration was within 20% of the target, the method of infusion was extended; intermittent dosing to extended, or continuous. If antibiotic concentrations were too high, dosing frequency was reduced by 25-50%.	Meropenem patients that received a high dose were prescribed significantly more antibiotic than the licensed dose group [p=0.03].<br><br>There was no difference in the number of treatment courses that achieved 100% ƒT>MIC. Licensed dose: n=10 (45.5%), high dose: n=15 (53.6%) [p=0.57].	Microbiological control was seen in 33/47 patients, 13/22 in the licensed dose group and 20/27 in the high dose group [p=0.12].<br><br>No statistical associations were made between microbiological resolution and achieving adequate antibiotic concentrations.	In meropenem patients, there was no difference in the duration of therapy between the groups, licensed dose group: 6.6+/-3.8 days, high dose group: 7.9+/-4.5 days [p=0.28].<br><br>In meropenem patients there was no difference in treatment failure [p=0.56] and length of stay in ICU [p=0.17]. There was a near significant difference in the length of hospital stay between licensed-dose (156.6 days +/- 185 days) and high dose (68.4 days +/-130.8 days) [p=0.06]. There were 2 deaths in the licensed dose group and 1 in the high dose group.<br><br>No excessive toxicity was seen in either group.<br><br>No statistical associations were made	Not specified

										between achieving adequate concentrations and clinical outcomes.	
Pea *et al*, 2017. [48]<br><br>Study looking at improving clinical cure in KPC-producing *Klebsiella pneumoniae* (KPC-Kp) through real-time dose adjustments of continuously infused meropenem	Patients with a KPC-Kp infection receiving meropenem, n=30.<br><br>Median Charlson co-morbidity index score was 2 (IQR: 2-3)	Meropenem, n=30.<br><br>Continuous infusion: 1.7g/24h - 13.2g/24h.	Antibiotic concentrations determined by HPLC-UV.<br><br>Median number of antibiotic concentration measurements: 4.	All patients received meropenem as a combination therapy, including: amikacin, ciprofloxacin, tigecycline, colistin, gentamicin, fosfomycin, ertapenem, rifampicin, and trimethoprim-sulphamethoxazole.<br><br>The number of concomitantly administered antibiotics was not associated with clinical cure.	Median estimated CrCl: 80.65 mL/min (IQR: 53.63 mL/min - 112.20 mL/min)<br><br>Analysis of effects of renal function on target attainment/clinical outcomes not reported.	All patients had a KPC-Kp infection.<br><br>Median MIC for meropenem was 32 mg/L (IQR: 4-64 mg/L).	A C_{ss} /MIC ratio of ≥1-4 was used as a pharmacological target.<br>Where KPC-Kp isolates had a MIC ≤16 mg/L a C_{ss} ratio target of ≥4 was used. Where isolates had a MIC of 32-64 mg/L a C_{ss} ratio target of 1-3 was used. Where isolates had a MIC >64 mg/L a C_{ss} ratio target of 1 was used, although stated as "sub-optimal".<br>Dose adjustments were based on antibiotic concentration, susceptibility of the pathogen and the patients pathophysiological data. No dose adjustment protocol specified.	Mean meropenem C_{ss} ranged from 15.6-143.0 mg/L across all patients.<br><br>Median number of dose adjustments: 1 (IQR: 1-2).<br>Median time to reach optimal antibiotic exposure: 2 days (IQR: 1.75 – 4 days), of assessable patients (n=24).	Not specified	Successful clinical outcomes seen in 73.3% of cases.<br>Median length of treatment was 14.0 days (IQR: 8.0 - 18.3 days).<br><br>Univariate analyses showed a significant association between clinical success and: C_{ss}/MIC of ≥1 (OR=10.556; 95% CI 1.612-69.122, p=0.014), C_{ss}/MIC of ≥4 (OR= 12.250; 95% CI 1.268 - 118.361, p=0.03), and Charlson co-morbidity index of ≥4 (OR=0.158; 95% CI 0.025 - 0.999, p=0.05).	Not specified
Cojutti *et al,* 2015. [49]<br><br>Study investigating the pharmacokinetics and pharmacody	Paediatric patients that had undergone HSCT treatment and were administered with	Meropenem, n=21.<br><br>A loading dose was administered prior to the continuous infusion.	Total plasma antibiotic concentrations determined by HPLC-UV.<br><br>Samples taken for antibiotic concentration	19/27 patients (70.4%) received concomitant antibiotics alongside meropenem. Co-administered	Median CrCl: 189.5 mL/min/1.73m^2 (IQR: 118.5-265.6 mL/min/1.73m^2).	Pathogens isolated: *E. coli* ESBL$^-$ n=4 (14.8%), ESBL$^+$ n=2 (7.4%); *P. aeruginosa* n=3 (11.1%); *K. pneumoniae* KPC$^+$ n=1	A C_{ss}/MIC ratio of 4-6 was targeted, where the MIC value is based on the EUCAST clinical breakpoint value for *Pseudomonas aeruginosa* and	Median meropenem C_{ss}: 29.8 mg/L (IQR: 19.2-41.42 mg/L). Following antibiotic concentration measurements dose reductions	Not specified	Out of patients where a pathogen was isolated (n=11) there was clinical cure in 9 (81.8%). Two patients died due to	Not specified

namics of continuously infused meropenem in paediatric haematopoietic stem cell transplant (HSCT) patients.	continuously infused meropenem guided by TDM, for a Gram-negative infection, n=21.	Median dose: 92.69 mg/kg/day (IQR: 67.8 – 130.7 mg/kg/day).	measurement ≥2 days of continuous infusion of the same dose, to reach steady-state. Frequency of sampling not specified.	antibiotics included amikacin, teicoplanin, tigecycline, linezolid, vancomycin, and colistin.	Median meropenem clearance: 2.3 mL/min/1.73m^2 (IQR: 1.4-4.4 mL/min/1.73m^2).	(3.7%); *E. coli* with porin - mediated resistance n=1 (3.7%); *Propionibacterium acnes* n=1 (3.7%); polymicrobial infections n=2 (7.4%); unidentified n=17(62.9%).	*Enterobacteriaceae* (2 mg/L).<br><br>Doses were adjusted to target C_{ss} of 8-12 mg/L.	were recommended in 54.5% of patients and dose increases were not recommended. In 11/27 (40.7%) patients meropenem C_{ss} was reassessed at least once (n=16) and further dose reductions were recommended in 8/16 (50%) of cases.		underlying malignancies.<br><br>No associations were made between clinical outcome and target attainment, or any other confounding variable.	
Cojutti *et al*, 2020. [50]<br><br>Study looking at the effects on treatment outcome of febrile neutropenia by adjusting continuously infused meropenem dosing regimens according to TDM result in oncohaematological patients	Oncohaematological disease patients receiving meropenem for febrile neutropenia , n=75	Meropenem, n=75<br>Empiric dose: 1g loading dose over a 30 min infusion followed by a continuous infusion of 1g q8h, over 8 hours. A continuous infusion of 500mg q6h was used if eCrCl <60 mL/min/1.73 m^3.	Plasma antibiotic levels determined by HPLC-UV.<br><br>Antibiotic levels assessed after 48-73h and then every 48-72h thereafter. Median number of antibiotic measurements per patient: 3, (IQR: 2-4).	51 patients (68%) received meropenem alongside a concomitant agent. Amikacin was administered to 12 (16%), 31 (41.3%) received an anti-Gram-positive agent, and 8 (10.7%) received both amikacin and an anti-Gram-positive agent. Antifungals were co-administered to 7 patients (9.3%).	Median estimated CrCl at baseline: 113.3 mL/min/1.73m^2 (IQR: 85.7 – 144.3 mL/min/1.73m^2).<br><br>Patients with ARC: n=37 (36.0%).	From the 75 patients 20 pathogens were isolated: *E. coli* n=9, meropenem MIC: ≤0.125 mg/L; *P. aeruginosa* n=4, meropenem MIC: ≤0.125-32 mg/L;<br>KPC-Kp n=1, meropenem MIC: 16 mg/L; *Citrobacter freundii* n=, meropenem MIC: ≤0.125; MRSA n=2; MRSE n=2; *Enterococcus faecium* n=1.	A C_{ss}/MIC ratio of 4-8 was targeted, where the MIC value is based on the EUCAST clinical breakpoint value for *Pseudomonas aeruginosa* and *Enterobacteriaceae* (2 mg/L).<br><br>Doses were adjusted to target C_{ss} of 8-16 mg/L. In pathogens with a meropenem MIC >32mg/L, where clinicians deemed appropriate, a high dose strategy was implemented where a C_{ss}/MIC of 1-3 was targeted. A maximum threshold of 100 mg/L was set.	Median meropenem C_{ss}: 12.7 mg/L (IQR: 9.7-17.6 mg/L). There were 46 dose adjustments out of 153 TDM reassessments, increases in 24 and decreases in 22.<br>No information to show that dose adjustments improved target attainment.	Not specified.	67/75 patients (89.3%) were cured. 14-day mortality was seen in 8/75 patients (10.7%).<br><br>From multivariate logistic regression analyses, 14-day mortality was significantly associated with ARC (OR 10.846, 95% CI 1.534-76.672, p=0.017) and HSCT (OR .086, 95% CI 0.008-0.36, p=0.044).<br><br>Out of patients with an isolated Gram-negative infection (n=15) 14 were cured, all 14 had a C_{ss}/MIC ratio of >1. The patient	Not specified.

										with a failed clinical outcome had a *P. aeruginosa* infection with a MIC of 32, and a C_{ss}/MIC ratio of 0.59.<br><br>No statistical associations were made between clinical outcome and meropenem concentration.	
Patel *et al*, 2012. [51]<br><br>Study observing the effects of TDM guided dosing regimens in burns patients<br><br>**, n=49	Burns patients, n=50<br><br>% TBSA: 17 +/- 13	Meropenem, n=1<br><br>Initial dose: 1g q8h	Plasma concentrations determined by HPLC-UV. Unbound concentrations estimated using published protein binding data.<br><br>Samples for antibiotic measurement taken after a minimum of 4 prior doses, to ensure PK was at steady-state. Samples were taken within 15 minutes of next dose.	Concomitant antibiotics not specified.	Serum creatinine concentration over the whole study group: 86 µmol/L +/- 30 µmol/L**<br><br>Analysis of effects of renal function on target attainment not performed.	Pathogens responsible for infections not specified.<br><br>Pathogen or suspected pathogen clinical breakpoint values from local antibiogram data were used for MIC values where possible, otherwise clinical breakpoint values from EUCAST data were used.	100% ƒT>MIC and was used as the pharmacological target, 100% ƒT>4xMIC was also used in the analysis.<br><br>If TDM results showed a concentration below the 100% ƒT>MIC target then dosing frequency was increased. If dosing frequency had already been increased then the infusion was extended. There was no protocol for antibiotic concentrations that were too high.	30 patients (60%) received a dose adjustment after initial antibiotic concentration result. Following dose adjustments all patients exceeded the minimum target.<br><br>In meropenem patients no patients reached the ƒT>MIC target, or the ƒT>4xMIC target.	Not specified	A positive clinical outcome was seen in all patients included in the study.<br><br>In patients where the ƒT>MIC target was achieved there was a significantly shorter length of antibiotic treatment, 4.2 +/- 1.1 days, versus 5.3 +/-2.3 days [p=0.03], than in patients where the target was not met.<br><br>When using the ƒT>4xMIC target there was not a statistically significant association, 4.1 +/- 1.1 days where the 4xMIC target was met versus	Not specified

										5.1 +/- 2.1 days; [p=0.17].	
Wong *et al*, 2018. [52]<br><br>TDM guided dose adjustment in critically ill patients<br><br>**, I.E.=272	Critically ill patients, n=330<br><br>Median APACHE II score: 22 (IQR: 16-27)	Meropenem, I.E.=97<br><br>Standard dose: 1g q8h	Unbound plasma concentrations determined by HPLC-UV.<br><br>Sampling undertaken after 4 prior doses, for PK to reach steady-state.<br><br>For intermittent dosing, a mid-point and trough sample was taken.<br><br>For continuous infusions, samples not taken until four half-lives of the antibiotic had passed.<br><br>103 patients (31.2%) had multiple TDM measurements	Patients with concomitantly administered antibiotics were included, there was no analysis into the effect of concomitantly administered antibiotics on outcomes.	Serum creatinine concentration: 76 μmol/L (IQR: 53-129 μmol/L)<br><br>Calculated CrCl: 101.5 mL/min (IQR: 9.1-163.0 mL/min)<br><br>On day of sampling 68 patients (13.8%) were undergoing CRRT.<br><br>ARC (CrCl >130 mL/min) was present in 192 patients (39.1%).<br><br>The presence of ARC (CrCl >130 mL/min) was significantly associated with failure to achieve PK target (OR 2.47-3.33, P<0.05)<br><br>Excessive antibiotic exposure was associated with decreased renal function (CrCl<50	Pathogens responsible for infections not specified.<br><br>Of the culture-positive samples, MIC data were available for 12 samples.<br><br>Where the MIC was not determined EUCAST clinical breakpoint values, for determined or suspected pathogens, were set as MIC values. Where no species was suspected or determined the highest MIC of a susceptible pathogen was selected.	Pharmacological targets of:<br>50% and 100% ƒT>MIC<br>50% and 100% ƒT>4xMIC<br>100% ƒT>10xMIC were set.<br><br>Where antibiotic concentrations were below 100% ƒT>MIC the frequency of dosing was increased by 25-50%. If antibiotic concentration was within 20% of target, infusions were extended, or continuous infusions were administered when antibiotic was at maximum dose according to products information.<br><br>When concentrations were >100% ƒT>10xMIC either the dose concentration was reduced by 50% or the dosing frequency was decreased by 25-50%.	Of patients where more than one antibiotic concentration was measured, there was no significant difference between initial TDM result and subsequent TDM result.<br><br>Of meropenem administered cases, 72.2% achieved the 100% ƒT>MIC target, 29.9% achieved 100% ƒT>4xMIC, and 11.3% met the 100% ƒT>10xMIC limit.<br><br>Extended infusions were associated with a decreasing probability of achieving 100% fT>MIC (OR 0.28 (95% CI 0.09-0.86); p=0.026)<br><br>Excluding the 50% ƒT>MIC target, the type of antibiotic was significantly associated with target attainment, [p<0.01]	Not specified	There was a positive clinical solution in 71.2% of cases.<br><br>There were 41 deaths across the study.<br><br>There was a significant association between negative clinical outcome and an abdominal source of infection (OR 7.60, (95%CI 2.39-24.17; P=0.001).<br><br>There was no association observed between patients that failed to achieve pharmacological targets and negative clinical outcomes in patients with a positive microbiological culture (100% fT>MIC: OR 0.88 (95% CI 0.40-1.94; p=0.74. 100% fT>4xMIC: OR 0.67(95% CI 0.29-1.55; p=0.35).	Not specified

					mL/min, OR 9.12 (95% CI 3.05-27.25); p<0.01. CrCl 51-90 mL/min, OR 3.21 (95% CI 1.10-9.41); p=0.03).						
Roberts *et al*, 2010. [53]<br><br>Study describing the effect of TDM guided dosing regimens in critically ill patients<br><br>**, n=185	Critically ill patients n=236<br><br>Patient condition not specified.	Meropenem, n=51<br><br>Empiric dose: 1g q8h	Total plasma concentrations determined by HPLC-UV, free antibiotic concentrations calculated using published protein binding data.<br><br>Antibiotic concentration measurement performed twice a week. For intermittently dosed patients, sampling was initiated at steady-state, after 4 previous doses, within 15 minutes of next dose. For patients under continuous infusion, samples were taken at stead-state, after 4-5 half-lives of antibiotic.	The study hospital uses single-therapy treatments, and only prescribed additional antibiotics in enrolled patients to treat other pathogens that were resistant to the beta-lactam prescribed.<br><br>There is no analysis to show how many patients were administered concomitant antibiotics, nor the effect of the concomitant antibiotics on outcome.	Mean serum creatinine concentration at the start of treatment: 111µmol/L +/- 91µmol/L.<br><br>ARC (CrCl >150-160 mL/min) was seen in 21/47 patients (44.7%) where 8-hr CrCl was taken.<br>Of the 21 patients displaying ARC, dose increases were needed in 16 of these patients (76.2%), and dose decreases were needed in 3 patients (14.3%), target attainment was seen in 2 patients (9.5%).<br><br>Renal dysfunction (>180µmol/L serum	Commonly isolated pathogens: MSSA (n=39), *Klebsiella* spp. (n=16), *Escherichia coli* (n=14).<br><br>MIC values were obtained from local antibiogram data, or where not available EUCAST data.	A pharmacological target of 100% ƒT>4-5xMIC was used.<br><br>If concentrations were below target then dosing frequency was increased by 25-50%. If concentrations were within 20% of the target then administration infusion was extended. When intermittent dosing was at maximum dose according to product then continuous dosing was initiated.<br><br>If concentrations were >100% ƒT>10xMIC then dose was decreased by 50% or dosing frequency was decreased by 25-50%.	In meropenem patients: dose maintained: 8 (16%), dose increased: 29 (57%), dose decreased: 14 (27%).<br><br>Of the 51 patients, including non-carbapenem treated patients, where a second sample was taken for TDM analysis 22 (43.1%) achieved the target concentration.	Not specified	A positive clinical resolution was seen in 206/236 of antibiotic courses.<br><br>There was no significant association between negative clinical outcome and subtherapeutic antibiotic levels (p=0.34), or increased serum creatinine concentration (p=0.34). The only factor significantly associated to negative clinical outcome was APACHE II score (p=0.05).<br><br>The mortality rate for patients with initial subtherapeutic antibiotic levels was 3.3%, compared to 8.2% who had an initial TDM within the target range, and 9.3%	Not specified

					creatinine) was seen in 10 patients, 9 of which needed dose adjustments. CVVHDF was being given to 36 patients during initial TDM sampling, 25 (69%) needing dose adjustment. Surgical drains were seen in 60 patients, of 52 not needing CVVHDF, 41 (79%) needed a dose adjustment.					who had an initial concentration above the target range. Factors predictive of mortality were APACHE II score ($p<0.01$) and increased serum creatinine concentration ($p=0.05$).	
Bricheux *et al,* 2019. [54] TDM use in imipenem treatment in hospitalised patients	Hospitalised patients with suspected or confirmed infections, n=300.<br><br>126 patients (42%) required intensive care.	Imipenem, n=300.<br><br>Empiric dose: 500mg q6h administered as a 15 min infusion. Occasionally administered as a 4 hour extended infusion.	Total plasma antibiotic concentrations determined by HPLC-UV.<br><br>TDM was performed on request and available on weekdays.	Not specified	Median CrCl at admission: 68.2 mL/min (IQR: 39-101 mL/min). Median CrCl at first TDM in all patients: 78 mL/min (IQR: 45-122 mL/min).<br><br>There was a significant difference in CrCl between patients with clinical failure (median CrCl: 63.3 IQR: 39-90 mL/min) and without (median CrCl: 82.3 (49-143 mL/min) at	The majority of infections were microbiologically confirmed, 194/300 (65%).<br><br>Commonly isolated pathogens were Enterobacteriaceae (n=121), and *Pseudomonas aeruginosa* (n=29).	No pharmacological target was specified.<br><br>Clinicians adjusted dose at their discretion.	Imipenem concentrations were low with some lower than the limit of detection. Median imipenem trough concentration from first TDM result was 3.2 mg/L (IQR: 1.7 – 6.5 mg/L). Measured concentrations following dose adjustment not specified.	Not specified.	Out of the 300 patients there was imipenem-related toxicity in 15. Of which 7 were considered possibly related, 7 likely related, and certainly related to imipenem. There was no statistically significant association between imipenem concentration and toxicity.<br><br>There was clinical failure in 88 patients (29%), of these 52 (60%) died.	Not specified

					time of first TDM, $p<0.001$.					Clinical failure was associated with pneumonia (OR 1.8, 95% CI 1.0-3.1 p=0.04) in univariate analyses. imipenem concentration was not significantly associated with clinical failure.	
Schoenberger-Arnaiz *et al* 2019. [55]<br><br>Determining the usefulness of TDM guided continuous infusions of piperacillin and meropenem<br><br>**, n=92	Critically ill patients, n=124<br><br>APACHE II score: 18.1 (+/- 7.5) **	Meropenem, n=32.<br><br>Empiric dose: Continuous infusion, 3g or 6g/24h	Total serum concentrations of meropenem determined by HPLC-UV, free antibiotic concentrations calculated using published protein binding data.<br><br>Antibiotic measurements taken twice weekly. In 49/93 (52.6%) only one measurement was available. 41/93 (44.1%) had two measurements and 3/93 (3.3%) had three.	Not specified	Mean meropenem clearance: 2.43 +/- 1.7 ml/kg/min.<br><br>There was a significant difference in meropenem clearance in patients requiring a dose adjustment and those requiring CRRT, compared to those not requiring a dose adjustment [$p<0.0001$]	Isolated pathogens treated with meropenem: *E. coli* (n=12), *P. aeruginosa* (n=1), other (n=1).<br><br>MICs were determined by broth microdilution, if necessary this was confirmed by an E-test. If a MIC could not be determined then an empiric MIC was set, for meropenem this was ≤4mg/L.	100% ƒT>4xMIC was used as the minimum pharmacological target.<br><br>Dose adjustment protocol not specified. Dose adjustments were determined by clinicians. Antibiotic concentrations were available to influence dosing regimens, dose adjustments were also carried out according to existing procedures.	Of the 41 meropenem cases, 26 did not need a dose adjustment and had normal renal function, (mean concentration of 20.7 mg/L +/-10.3 mg/L). There were 6 cases requiring a dose adjustment, with no CRRT needed, (mean meropenem concentration of 65.5 mg/L +/- 25.4 mg/L). There were 9 cases requiring CRRT, (mean meropenem concentration of 39.8 mg/L +/- 16.4 mg/L).	Not specified	No difference in mortality between patients where target concentrations were reached compared to not.<br><br>Of patients where there was a dose adjustment, mostly reductions, all-cause mortality was 25%. In those where there was no dose adjustment there was a 10.5% all-cause mortality.	Not specified
Gatti, *et al*, 2021. [56]<br><br>TDM carried out in critically ill patients with Gram-	Critically ill patients (n=116).<br><br>n=62 (53.5%) had septic shock.	Meropenem, n=52.<br><br>Empiric dose: loading dose of 2g over a 2h infusion.	Total meropenem concentrations determined by LC-MS/MS.<br><br>Samples collected in	Concomitant antibiotic treatment seen in n=23 patients, (19.8%).	Median CrCl: 74.5 mL/min/1.73 m^2, (IQR: 39.8 – 102 mL/min/1.73 m^2).	Isolated Gram-negative pathogens: *Klebsiella pneumoniae* (n=35, 25.2%); *Pseudomonas aeruginosa*	No target set, doses were adjusted to treating clinicians' discretion.	Median meropenem C_{SS}/MIC: 32.4 (IQR: 3.9 - 211.3).	Microbiological failure in n=13 meropenem patients and n=26 in total, 22.4%.	Median length of therapy: 10 days (IQR: 6-14 days).	Resistance developed in n=20, 17.2%.<br><br>In univariate analysis resistance development

negative infections<br><br>**, n=64	n=101 (87.1%) required mechanic ventilation. n=26 (22.4%) received CRRT.	Maintenance dose infused continuously every 6-8h.<br><br>Median meropenem dose 4g/24h (IQR: 2-4g).	first 72h of treatment. Results available within 6h from blood collection.	Colistin, n=9; fosfomycin n=8; tigecycline, n=5; ciprofloxacin, n=1.	ARC: n=13, (11.2%).	(n=33, 23.7%); *Escherichia coli* (n=27, 19.4%); *Enterobacter* spp. (n=14, 10.1%); *Proteus mirabilis* (n=7, 5%); *Acinetobacter baumannii* (n=6, 4.3%); *Serratia marcescens* (n=3, 2.2%); others (n=14, 10.1%).	No dose adjustment protocol reported.		Median time to microbiological failure: 11.5 days (IQR: 8.3-14). *<br><br>In univariate analysis microbiological failure was associated ($p<0.05$) with: ARC ($p=0.007$); Septic shock ($p=0.003$); Pneumonia ($p=0.023$); *P. aeruginosa* ($p=0.002$); *E. coli* ($p=0.048$); *A. baumannii* ($p=0.021$); C_{SS}/MIC ≤ 5 ($p<0.001$); combination therapy ($p=0.036$).<br><br>In multivariate analysis microbiological failure was associated ($p<0.05$) with: *P. aeruginosa* ($p=0.036$); C_{SS}/MIC ≤ 5 ($p<0.001$).		was associated ($p<0.05$) with: ARC ($p=0.007$); Septic shock ($p=0.003$); Pneumonia ($p=0.023$); *P. aeruginosa* ($p=0.002$); *E. coli* ($p=0.048$); *A. baumannii* ($p=0.021$); C_{SS}/MIC ≤ 5 ($p<0.001$); combination therapy ($p=0.036$).<br><br>In multivariate analysis resistance development was associated ($p<0.05$) with: *P. aeruginosa* ($p=0.036$); C_{SS}/MIC ≤ 5 ($p<0.001$).
Aldaz *et al*, 2021. [58]<br><br>Meropenem TDM carried out in critically ill patients	Critically ill patients, n=173 (n=154 after pairing).<br><br>SOFA score on admission, after pairing:	Meropenem, n=154 after cohort pairing.<br><br>Median daily dose, TDM group: 3g (range: 1-3g); standard care	Meropenem serum concentrations determined using high-resolution LC-UV.<br><br>TDM initiated after third	Concomitant antibiotics were reported: vancomycin, quinolones, linezolid, aminoglycosides, trimethoprim/Sulphamethoxa	After pairing, median CrCl: TDM group: 56.1 mL/min (range: 17.38-135.05); standard care group: 51.89 (10.38-	Commonly isolated pathogens: *E. coli* (TDM: 7.03%, standard care: 21.54%); *P. aeruginosa* (TDM:11.72%, standard care: 4.62%);	Pharmacological target of 100% ƒT>4-5xMIC was used.<br><br>No formal dose adjustment protocol published.	There were adjustments in the TDM group in 51 patients (66.23%), reducing the daily dose in 46 patients.<br><br>In the TDM group n=46 (59.74%)	Microbiological eradication; TDM group: n=66 (85.70%), standard care group: n=62 (80.50%), $p=0.390$.	There were significant differences between the TDM group and standard care group in: Number of patients with ≥80% reduction	Not specified.

	TDM: 4 (range:0-14)<br>Standard care: 4 (0-12)	group: 3g (range: 1-4g).	dose. C_{max} and C_{elim} (3 hours after first sample) were measured and PK parameters estimated.	zole, daptomycin, metronidazole, azithromycin/clarithromycin, colistimetate, ampicillin all used.<br>No significant differences in concomitant antibiotics between TDM and standard care groups, p>0.100.	153.13); p=0.555.	*Staphylococcus* spp. (TDM:16.41%, standard care: 23.85%); *Klebsiella* spp. (TDM: 8.59%, standard care: 6.15%).<br><br>E-test used to determine MIC values. If no MIC available, then a 1 mg/L susceptibility cut-off was used based on local microbiological MIC data.<br>Median MIC: 0.25 mg/L (range: 0.006-2 mg/L).		patients met the target.<br>Median C_{min}/MIC: 24.3 (range: 0.27-46).<br><br>No data was recorded for the standard care group.		in PCT, p=0.02; PCT levels after treatment, p=0.0130; % reduction of PCT, p=0.004; fever, p=0.036; CRP normalisation, p=0.003; PCT normalisation, p=0.006; hospital length of time, p=0.007.<br><br>There were no statistical differences between TDM and standard care in: adverse effects; leukocytes; neutrophils; SOFA score on discharge; ICU length of time; readmission after 30 days; in-hospital mortality; 14 day mortality.	
Al-Shaer *et al*, 2020. [57]<br><br>TDM of β-lactams and associated health outcomes.<br><br>**, n=174	Critically ill patients, n=206<br><br>Median SOFA score: 5 (IQR: 2-8)<br><br>Median APACHE II score: 17 (12-22)	Meropenem, n=32<br><br>Median daily dose: 4g (IQR: 1-12)	Total antibiotic concentrations determined through LC-MS/MS. Free concentrations calculated using published protein binding data.<br><br>Peak and trough concentrations determined.	Patients receiving concomitant antibiotics:<br><br>Aminoglycosides: 53 (26%)<br><br>Colistin: 8 (4%)<br><br>Fluoroquinolone: 18 (9%)	Baseline median CrCl: 90.9 mL/min (IQR:52.0-136.4 mL/min)	Commonly isolated pathogens: *P. aeruginosa* (n=91); *K. pneumoniae* (n=41); *E. coli* (n=29); *Enterobacter cloacae* complex (n=30); MSSA (n=15)<br><br>MICs were measured by	100% ƒT>MIC and 100% ƒT>4xMIC were used as pharmacological targets.<br><br>Dose adjustment protocols were not specified.	100% ƒT>MIC target attainment: Meropenem: 29 (90.6%)<br><br>100% ƒT>4xMIC target attainment: Meropenem: (81.3%)<br><br>Of 12 that received a dose adjustment 7 had an additional TDM measurement. Of those 4 (57%) met	Microbial eradication was significantly associated with 100% ƒT>MIC from the first TDM sample, p=0.0476.	100% ƒT>MIC from the first TDM sample was significantly associated with clinical cure (p=0.0303), SOFA score, and days to measuring concentration.<br><br>Days to measuring concentration was significantly	Emergence of new resistance was significantly associated with a first TDM sample of 100% ƒT>4xMIC, p=0.0043.

						VITEK 2 or Etest.<br><br>Meropenem median MIC: 0.375 (IQR:0.25-16)		the 100ƒT>MIC target, and 2 met the 100%ƒT>4xMIC target.		associated with clinical cure, ICU length of stay, and mortality.	
Scharf *et al*, 2020. [59]<br><br>Determining optimal β-lactam concentrations for critically ill patients<br><br>**, n=21	Critically ill patients, n=55<br><br>Median SOFA score: 10 (min-max: 3-19)<br><br>Median APACHE II score: 25 (min-max:12-251)	Meropenem, n=34<br><br>Dose not specified	Meropenem concentrations determined by LC-MS/MS.<br><br>Patients with ≤2 antibiotic concentration measurements excluded.	Patients with concomitant antibiotics were excluded.	Baseline median CrCl: 79 mg/L (min-max: 4-200 mg/L)<br><br>Baseline creatinine: 0.8 mg/dL (min-max: 0.4-5.3 mg/dL)<br><br>Creatinine clearance was higher in group 1 and lower in group 3, compared to group 2.	Isolated pathogens: *P. aeruginosa* (MIC: 16 mg/L); *E. coli* (8 mg/L); *Klebsiella oxytoca* (MIC: 8mg/L); *Serratia marcescens* (MIC: 8 mg/L); *Enterococcus faecalis/faecium* (4 mg/L); *Achromobacter xylosoxidans* (MIC: 8 mg/L); *Proteus mirabilis* (MIC: 8 mg/L); *Shewanella putrefaciens* (MIC: 8 mg/L); *Streptococcus anginosus* (MIC: 8 mg/L). Based on EUCAST data.	100% ƒT>MIC<br>100% ƒT>4xMIC<br><br>Dose adjustments were at clinicians' discretion.<br><br>Subgrouped as Group 1: <100% ƒT>MIC<br>Group 2: 100% ƒT>1-4xMIC<br>Group 3: >100% ƒT>4xMIC	42.7% of meropenem trough TDM samples measured <100% ƒT>MIC<br><br>36.5% of meropenem trough TDM samples measured 100% ƒT>1-4xMIC<br><br>20.8% of meropenem trough TDM samples measured >100% ƒT>4xMIC	CRP significantly decreased in groups 2 and 3 compared to group 1. There was no difference between group 2 and 3.<br><br>No difference was seen in change in interleukin-6	No significant association in renal change or neurological disorders.<br><br>Group 3 (57.1%) had significantly higher mortality than group 1 (8.3%) and group 2 (17.6%). Group 1 and 2 were not different.	Not specified.

Abbreviations: APACHE II, Acute Physiology and Chronic Health Evaluation II; ARC, Augmented Renal Clearance (>130 mL/min/m^2); CNS, Central Nervous System; (e)CrCl; (estimated) Creatinine Clearance; CRP, C-Reactive Protein; CRRT, Continuous Renal Replacement Therapy; C_{SS}, Steady-state concentration; CVVHDF, Continuous Venovenous Haemodiafiltration; EUCAST, European Committee on Antimicrobial Susceptibility Testing; HSCT, Haematopoietic stem cell; I.E., Infection Episodes; ICU, Intensive Care Unit; (H/UP)LC, (High/Ultra-Powered Liquid Chromatography); MIC, Minimum Inhibitory Concentration; MS/MS, tandem Mass Spectrometry; PCT, procalcitonin; TBSA, Total Body Surface Area; SOFA, Sequential Organ Failure Assessment; UV, Ultra-Violet Spectroscopy; **, study included non-carbapenem antibiotics.

Supp. Table 3 – Study characteristics of clinical case studies where TDM has been used to guide carbapenem therapy

Reference	Patient Condition	Carbapenem	TDM measurement	Concomitant Antibiotics	Renal Function	Pathogen and MIC	Pharmacological Target	Pharmacological Target Attainment	Microbiological Resolution	Clinical Resolution	Emergence of Antimicrobial Resistance
Gunasekaran, K *et al,* 2018. [61]	Septicaemic melioidosis	Meropenem<br><br>1g q8h – as a 3hr infusion	TDM method not specified	Co-trimoxazole every 12 hours	Serum creatinine: 0.88 mg/dL	*B. pseudomallei* Meropenem MIC: 0.75 mg/L	4xMIC was used as a pharmacological target	1 week TDM results showed meropenem levels of 1.1mg/L.<br><br>Dosing regimen subsequently increased to 2g q8h, as 3hr extended infusions, after a 2g bolus dose.	Yes, 48 hours following dose adjustment culture conversion was achieved.	Yes, following dose adjustment patient improved clinically and was discharged.	Not specified.
Lonsdale, DO *et al,* 2013. [62]	Ventriculitis in critically ill patient	Meropenem<br><br>2g q8h	Antibiotic concentrations were determined from plasma and CSF samples using HPLC.	Vancomycin, 3.5g every 24hrs continuous infusion after 2g loading dose.	Creatinine Clearance (CrCl): 375 mL/min/1.73^2	*Klebsiella pneumoniae* Meropenem MIC: 2 mg/L<br><br>(*Staphylococcus epidermidis* Vancomycin MIC: 2 mg/L)	>MIC was used as a pharmacological target.	The initial meropenem dosing regimen concentrations were undetectable at <0.1mg/L on day 5 of therapy, in both plasma and CSF. Meropenem dosing frequency was increased to 2g q6h. Which resulted in plasma trough concentrations of 2.0 and 2.4 mg/L on days 6 and 7, respectively. And 1.8 and 3.0 mg/L in CSF on days 6 and 7, respectively.	*K. pneumoniae* was isolated up to day 27 of treatment. Meropenem therapy was continued until day 37.	The patient improved and was discharge from ICU.	Not specified.
Udy AA, *et al*, 2010. [63]	P1: Ventriculitis following external ventricular drain treatment.	P1: Meropenem 1g q8h<br><br>P2: Meropenem 1g q8h	Serum trough levels of antibiotics were determined. Method of TDM not specified.	P1: No concomitant antibiotics specified<br><br>P2: Vancomycin was co-administered	P1 UO: 1 mL/kg/h. Serum creatinine (SCr): 58 µmol/L. Estimate glomerular filtration rate	P1: *Enterobacter* spp. Meropenem MIC: 2 mg/L<br><br>P2: *Klebsiella pneumoniae*	P1: Target not specified<br><br>P2: Target concentration 8-10 mg/L, 4-5xMIC	P1: Initial trough serum concentrations were below limit of detection (<5 mg/L). Dosing regimen was increased to 2g	P1: Microbiological resolution was confirmed through negative CSF cultures.	P1: Patient condition improved and was discharged from ICU.<br><br>P2: Patient condition	P1: Not specified<br><br>P2: Not specified

	P2: Intra-abdominal sepsis				(eGFR): >90 mL/min measured 8hr CrCl: 224 mL/min.<br><br>P2 UO: >1.2 mL/kg/h. SCr: 56 µmol/L Measured 8-hr CrCl: 206 mL/min	meropenem MIC: 2 mg/L) (*Enterococcus faecium* Vancomycin MIC: 1 mg/L)		q8h, but still undetectable in TDM result.<br><br>P2: Initial TDM result was undetectable (<5 mg/L). Dosing regimen was increased to 1g q6h but remained undetectable (<5 mg/L). Dosing frequency was further increased to 1g q4h where TDM result showed a serum trough concentration of 10 mg/L, meeting the target.	P2: Microbiologic al improvement was observed.	improved and was discharged from ICU.	
Hayashi Y, *et al,* 2013.[64]	P1: Mediastinitis<br><br>P2 and P3: Not treated with Carbapenems	Ertapenem 1g q12h	Plasma samples were taken after treated had reach steady-state. Antibiotic levels were determined by HPLC-UV.	Not specified	Measured 8h CrCl: 238 mL/min	*Enterobacter cloacae* Ertapenem EUCAST susceptibility breakpoint: 1 mg/L	ƒT>MIC was used as a pharmacological target.	Initially TDM result on day 6 of therapy was undetectable (<1 mg/L). On day 8 total concentrations were 3 mg/L, ertapenem is highly protein bound, estimated unbound concentration was 0.3mg/L. Dosing regimen was increased to 1g q8.h. On day 14 TDM result showed an estimated unbound level of 0.6 mg/L, and so the infusion was extended to 4h, from 30 mins. On day 17 ertapenem	Not from Ertapenem therapy.	Patient did not improve from Ertapenem therapy. Subsequent meropenem therapy improved patient condition.	Estimated unbound ertapenem concentrations were below the susceptibility breakpoint until day 17 by then cultures of *E. cloacae* showed resistance to ertapenem (MIC: 4mg/L).

								estimated unbound concentrations reached 1.2mg/L.			
Wu, YE *et al*, 2020. [65]	Neonatal sepsis, suspected neonatal meningitis	Meropenem 20 mg/kg q12h, increased to 40 mg/kg q12h after suspicion of meningitis	Plasma concentrations were determined through reverse phase HPLC	Vancomycin, 15 mg/kg single dose.	SCr: 80 µmol/L	*K. pneumoniae* MIC: 8mg/L	70% ƒT>MIC was used as the pharmacological target.	Target attainment was estimated using Monte Carlo simulations from TDM results. It was 99.2% probably that the target was met. The regimen was maintained	Negative blood cultures were observe.	Clinical signs and symptoms of infection improved.	Not specified
Oda, K *et al,* 2019. [66]	Pneumonia	Doripenem<br><br>3g/24hr continuous infusion	Unbound serum concentrations were determined by HPLC-UV	Tobramycin, 2.7 mg/kg/day, administered every 24 hours	CRRT, flow rate: 650 mL/h	*Pseudomonas aeruginosa* Doripenem MIC: 8 mg/L	100% ƒT>4xMIC was used as the pharmacological target	TDM result showed that unbound doripenem concentration was at 47.8 mg/L after 20 hours of treatment initiation, and 33.6 mg/L after 111 hours of treatment, within target.	Not specified	Patient condition improved and was discharged from ICU	Not specified
Troger, U *et al,* 2012. [67]	P1: Neutropenia<br><br>P2: Haemoptysis	P1: Meropenem, 1h q8h<br><br>P2: Meropenem, 1g q8h	Plasma concentrations were determined by HPLC-UV	P1: Piperacillin/Tazobactam was initially started before moving to meropenem. Vancomycin was co-administered with meropenem.<br><br>P2: Vancomycin, at 1g q24h, or 1g q12h	P1: SCr: 68 µmol/L. UO: 2.5mL/kg/h Estimated creatinine clearance: 138 mL/min.<br><br>P2: SCr: 46 µmol/L. UO: 1.5 mL/kg/L. Estimated creatinine clearance: 125 mL/min.	P1: *Staphylococcus aeureus* Meropenem MIC: 2 mg/L.<br><br>P2: *Klebsiella oxytoca* MIC not specified	60% ƒT>4-5xMIC was used as a pharmacological target.<br><br>Meropenem target concentration: 4-10 mg/L	P1: Initial TDM result demonstrated that meropenem concentration was below 4 mg/L. Dosing was increased to 2g q8h, however trough concentrations were below the target. The dosing regimen was further increased to 2g q4h.<br><br>P2: Initial TDM result demonstrated meropenem concentration of	P1: not specified<br><br>P2: not specified	P1: Signs and symptoms of infection improved.<br><br>P2: Patient condition improved and discharge from ICU.<br><br>High doses of meropenem were well tolerated with no signs of toxicity.	P1: Not specified<br><br>P2: Not specified

								<4 mg/L. The dosing regimen was increased to 1g q6h, and meropenem remained undetectable. The dosing regimen was further increased and TDM demonstrated a trough concentration of 8.4 mg/L. Within the target range.			
Cotta, MO *et al,* 2015. [68]	Severe burns patient	Meropenem, 2g q8h.	TDM method not specified	Piperacillin-Tazobactam was initially started, 4.5g q6h. Ciprofloxacin was later co-administered, 400mg q8h. Upon subsequent resistance to ciprofloxacin therapy was changed to gentamicin 600mg q24h. Amikacin, 1.5g q24h, was concomitantly administered with meropenem.	SCr: <30µmol/L Estimated CrCl: >200 ml/min 8-h urine measured CrCl: 129 mL/min/1.73m^2	*Escherichia coli, Klebsiella oxytoca, Enterococcus faecalis* from blood culture. *Pseudomonas aeruginosa, Enterococcus faecalis* from wound swab. *P. aeruginosa* meropenem MIC: 8mg/L.	100% ƒT>4xMIC, 32 mg/L.	The day-3 trough TDM for meropenem was 3.2 mg/L. Infusions were extended to 4 hour infusions. On day 7 meropenem trough concentrations were 5 mg/L. Meropenem dosing was increased to 2g q6h, as 3hr infusions. A trough concentration of 12 mg/L was then measured.	*P. aeruginosa* infection persisted.	Not specified	*Pseudomonas aeruginosa* acquired resistance to meropenem (MIC: >16mg/L)
Cojutti, PG *et al* 2018. [69]	Intracerebral haemorrhage patient with external ventricular drain	Meropenem, continuous infusion 500mg every 6 hours	Antibiotic concentrations were determined by HPLC-UV.	Cefotaxime 2g q8h was initially started. Therapy escalated to meropenem with linezolid concomitantly administered, 600mg q12h.	CrCl was measured on day 16: 131.0 mL/min, and on day 18: 160 mL/min<br><br>Meropenem clearance: 21.8 L/h	Not specified	Meropenem concentrations of 8-16 mg/L were targeted.	Initial TDM result showed meropenem concentrations were subtherapeutic as 3.82 mg/L. The meropenem dosing regimen was increased to 1.5 g every 6h by continuous	Not specified	Clinical improvement followed target attainment, signs of infection (CRP and PCT levels) decreasing.	Not specified

								infusion to reach the 8-16 mg/L target.			
Afaneh, CI *et al* 2012. [70]	Ventilator associated pneumonia in patient with acute kidney injury	Meropenem, 1g q8h in 3h extended infusion. Increased to 6g/24h continuous infusion	Total serum antibiotic concentrations were determined by HPLC. Unbound concentrations were estimated using published protein binding data.	Polymyxin B, 90mg q12h	SCr: 2.5 mg/dL eGFR: 25 mL/min	*Pseudomonas aeruginosa*, meropenem MIC: 4 mg/L	Steady state meropenem concentrations of >16 mg/L were targeted.	Target attainment was achieved with 6g/day continuous infusion therapy. Post-operative day 23 showed an unbound meropenem concentration of 32.83 mg/L. Days 24, 25, and 26 showed unbound meropenem concentrations of 21.96 mg/L, 18.26 mg/L and 15.93 mg/L, respectively. There was no change to dosing regimen from TDM result as patient was responding positively.	Not specified	The patient's condition improved and was discharged from hospital.	BAL samples showed multi-drug resistant *P. aeruginosa*.
Taccone FS, *et al* 2012. [71]	Ventilated associated pneumonia	Meropenem, 1g q8h	Total serum antibiotic concentrations were determined by HPLC-UV.	Ciprofloxacin, 400mg q8h. Ceased on day 6.<br><br>Colistin $6x10^6$ IU q12h, was co-administered from day 6.	SCr: 2.7 mg/dL	Multidrug resistant *Pseudomonas aeruginosa*, meropenem MIC: 2 mg/L	40% T>4xMIC	TDM result on day 2 showed a 37% T>4xMIC. On day 5 there was 39% T>4xMIC. Meropenem dosing was increased to 2g q8h over a 3h extended infusion. With this regimen, there was 39% T>4xMIC seen after pathogen was observed to be more resistant. The meropenem dosing regimen was increased to 3g q6h 3h extended infusion, after pathogen	Not specified	After target attainment patient condition improved. Resolution of signs of infection and patient was discharged from ICU on day 43.	A BAL sample on day 6 showed that *P. aeruginosa* meropenem susceptibility had decreased to a MIC of 4 mg/L. *P. aeruginosa* was later observed to be more resistant to meropenem, MIC: 8 mg/L.

								was seen to have become more resistant (MIC: 8mg/L). This resulted in a 51% T>4xMIC.			
Pea F, *et al* 2011. [72]	Severe cellulitis	Meropenem, ranging from 250mg q8h as a 6hr extended infusion to 500mg q4h as a continuous infusion	Method of TDM not specified	Daptomycin, ranging from 1.2g q48h as 30min infusions to 1.2 q36h as 30min infusions.<br><br>On day 29 therapy switched to amoxicillin/clavulanate and levofloxacin.	Renal failure	Not specified	Not specified	Dose adjustments were guided by TDM result. Target attainment not specified.	Not specified	Significant clinical improvement was seen from the TDM guided meropenem and daptomycin treatment.	Not specified
Stewart, A *et al* 2015. [73]	Cervical spine osteomyelitis	Meropenem continuous infusion 6g/24h.<br><br>Ertapenem continuous infusion 2g/24h.	Method of TDM not specified.	Days 1-27: tigecycline and vancomycin.<br>Days 28-42: colistin, amikacin, rifampicin, and fusidic acid.<br>Days 42-57: Amikacin (with meropenem).<br>Days 58-91: Fosfomycin (with ertapenem)	When receiving colistin, amikacin, rifampicin and fusidic acid eGFR: >90-52 mL/min/1.73m^2.<br><br>When receiving meropenm eGFR: >90 mL/min/1.73m^2.	Multidrug resistant *Enterobacter cloacae* and *Staphylococcus epidermidis*.	≥40% ƒT>MIC<br><br>Meropenem MIC 32 mg/L.<br><br>Ertapenem MIC: 1 mg/L	Average meropenem steady-state unbound concentration at 3.27g24h was 27 mg/L, at 4.8g/24h concentrations were measured at 28 mg/L, and 24.75 mg/L at 6g/24h.<br><br>Average ertapenem unbound concentrations at 2g/24h dosing was 6.6 mg/L and 6 mg/L at 1.8 g/24h.	Not specified	Clinical improvement was seen in response to ertapenem treatment.	Not specified
Cies, JJ *et al* 2017. [74]	Non-cystic fibrosis bronchiectasis, patient receiving CRRT	Doripenem, initially dosed at 15 mg/kg q6h. Moved to continuous infusion of 2.5 mg/kg/hr.	Total serum antibiotic concentrations were determined by LC-MS/MS.	Vancomycin and cefepime. Azithromycin, ethambutol, and rifampicin.	Developed acute kidney injury. Serum creatinine: 0.68 mg/dL.	Methicillin-resistant *Staphylococcus aureus*	≥40% ƒT>MIC. Where the MIC for doripenem was estimated at <4 mg/L.	After switching to a 2.5 mg/kg/r continuous infusion of doripenem serum concentrations were measured at 4.01 mg/L.	Not specified	There was no clinical improvement observed and on day 10 the patient died.	Not specified

De Keukeleire, S *et al* 2016. [75]	Necrotising enterocolitis	Meropenem, initially administered at 20 mg/kg q8h as 30 minute infusions. Increased to 30 mg/kg q8h as a 30 minute infusion. Further increased to 40 mg/kg q8h as a 4 hour infusion.	Serum antibiotic concentrations were determined by HPLC-UV	Cefotaxime, vancomycin, and metronidazole administered prior to meropenem treatment. Amikacin and fluconazole concomitantly administered with meropenem. Ciprofloxacin and metronidazole were administered after the meropenem treatment.	Not specified	*Enterobacter cloacae*	50% T>MIC<br><br>MIC: Initially 1mg/L, increased to 6 mg/L.	TDM result from initial meropenem dosing regimen showed undetectable levels of meropenem. TDM result showed a fast metabolization of meropenem when administered at 30 mg/kg 30 min infusion with concentrations decreasing from 54 mg/L at 0 mins after infusion to 28 mg/L at 20 mins. After switching to the 40 mg/kg q8h over a 4h infusion dose, meropenem concentrations were on average at 26.2 mg/L over the 8 hour dosing period.	*E. cloacae* was eradicated, confirmed by repeat negative cultures.	Clinical improvement in response to treatment. Patient was discharged in a stable condition.	*E. cloacae* MIC increased from 1 mg/L to 6 mg/L. This was not yet at a resistant MIC and tested negative to molecular screening for producing carbapenemases.
Liebchen, U *et al* 2020. [76]	Pulmonary exacerbation in patient with cystic fibrosis. Lung transplant.	Meropenem, initially administered 2g q8h. Switched to 6g/24h continuous infusion. Increased to 8g/24h continuous infusion.	Method of TDM not specified.	Initiated on cefepime before switching to meropenem. High dose tigecycline and high dose sulbactam were administered following transplant. Colistin was prophylactically administered.	Elevated creatinine clearance, CrCl: 156 mL/min.	MDR *Pseudomonas aeruginosa*.<br><br>MDR *A. baumannii* cultured from donors lung.	Pharmacological target not specified.<br><br>*A. baumannii* meropenem MIC: 32 mg/L.	Initial 2g q8h dosing regimen resulted in insufficient meropenem concentrations of 1.22-2.13 mg/L. 6g/24h continuous infusion resulted in meropenem levels between 17.3 mg/L and 23.2 mg/L. 8g/24h continuous infusion resulted in average meropenem levels of 39.6 mg/L.	Not specified.	Clinical improvement was seen, with the patient exhibiting no signs of infection and the patient was discharged from ICU to a general ward.	Not specified.
Goutelle, S *et al*, 2021.[77]	Prosthetic joint infection	Ertapenem<br><br>P1: 1g/24h	LC-based method.	Not specified.	P1: 118 mL/min<br>P2: 90 mL/min<br>P3: 61 mL/min	P1: *E. cloacae*, MIC: 0.38 mg/L MIC initially not	100% ƒT>10-20x MIC, to achieve bone penetration.	P1: MIC was not initially available. MIC later	Not specified.	P1: Treatment failure	P1: Secondary acquired resistance.

		P2: 1g/24h<br>P3: 1g/24h<br>P4: 1g/12h<br><br>Administered subcutaneously.			P4: 63 mL/min	available, later measured at 0.38 mg/L<br>P2: *E. cloacae*, MIC: 0.064 mg/L<br>P3: *E. coli* MIC: ≤0.5 mg/L – assumed MIC, later measured at 0.032 mg/L<br>P4: *E. asburiae* MIC: 0.032 mg/L	P1 target: 7.6 mg/L<br>P2 target: 1.28 mg/L<br>P3 target: 10 mg/L – assumed MIC,<br>P4: *E. asburiae* MIC: 0.032 mg/L	measured at 0.38 mg/L. Dose failed to reach 10-20x target of 7.6 mg/L<br><br>P2: Target achieved 100% of the time<br><br>P3: Target achieved 100% of the time<br><br>P4: Target achieved 100% of the time		P2: Treatment Success<br><br>P3: Treatment Success<br><br>P4: Treatment stopped after COVID-19 diagnosis	P2: No emergence of resistance<br><br>P3: No emergence of resistance<br><br>P4: No emergence of resistance
Legg, A *et al,* 2020. [78]	P1: Breast cancer patient with pneumonitis<br><br>P2: Total rhinectomy for nasal squamous cell carcinoma patient.<br><br>P3: Osteomyelitis patient with renal impairment, type-2 diabetes, chronic foot wounds, hypertension.<br><br>P4: Meningoencephalitis secondary to mastoiditis.	Meropenem<br><br>P1 continuous infusion: 6g/24h.<br><br>P2: Continuous infusion 6g/24h.<br><br>P3: Continuous infusion 3g/24h.<br><br>P4: 2g IV q8h, 2g IV q6h, continuous infusion 10g/24h.	Plasma concentrations were determined by HPLC.	P1: trimethoprim/sulphamethoxazole<br><br>P2: Vancomycin.<br><br>P3: Clindamycin, ciprofloxacin, piperacillin/tazobactam.<br><br>P4: Not specified	P1 CrCl: 110 mL/min<br><br>P2 CrCl: 189 mL/min<br><br>P3 CrCl: 54 mL/min.<br><br>P4 CrCl: 262 mL/min.	P1: *Nocardia* infection CLSI breakpoint: 8 mg/L<br><br>P2: *Pseudomonas aeruginosa* resistant to piperacilliln/tazobactam and cefepime. *Enterobacter cloacae* meropenem MIC: 2 mg/L.<br><br>P3: *Pseudomonas aeruginosa,* MIC: ≤2 mg/L<br><br>P4: *Bacteroides fragilis* MIC: 0.032 mg/L, *Alcaligenes faecalis* 2 mg/L.	100% ƒT≥4xMIC	P1: Target was not met. Concentrations were ≥2x MIC after switching to continuous infusion. One TDM result showed a low result due to an error in the drug delivery system.<br><br>P2: After switching to continuous infusion, target was met.<br><br>P3: After switching to continuous infusion, target was met.<br><br>P4: After switching to continuous infusion, target was met.	P1: Not specified.<br><br>P2: Infection cured.<br><br>P3: Not specified.<br><br>P4: Infection resolved.	P1: Clinical cure. No adverse effects. 26 days of treatment.<br><br>P2: Clinical cure. 14 days of treatment, no meropenem-associated adverse effects.<br><br>P3: Clinical cure. 60 days of treatment.<br><br>P4: Clinical cure. Infection symptoms resolved. No meropenem adverse effects observed.	P1: Not specified<br><br>P2: Not specified.<br><br>P3: Not specified.<br><br>P4: Not specified.

Cojutti, P G *et al*, 2022. [79]	Severe SARS-CoV-2 infection. VAP.	Meropenem<br><br>Continuous infusion 6g/24h (1.5g/6h).	Method not specified.	Fosfomycin.	Continuous venovenous haemodiafiltration (CVVHDF)	XDR *Klebsiella pneumoniae,* meropenem MIC: 16 mg/L	C_{SS}/MIC: 1-4	Concentrations were maintained within target, following a number of dose adjustments.	Consecutive blood cultures confirmed eradication.	Clinical success.	Not specified.

Abbreviations: BAL, bronchoalveolar lavage; CrCl, creatinine clearance; CRRT, continuous renal replacement therapy; C_{SS}, steady-state concentration; CSF, cerebrospinal fluid; CVVHDF, continuous venovenous haemodiafiltration; EUCAST, European Committee on Antimicrobial Susceptibility Testing; (e)GFR, (estimated) glomerular filtration rate; ICU, intensive care unit; IV, intravenous; (HP)LC, (high-powered) liquid chromatography; MIC, minimum inhibitory concentration; M/XDR, multi/extensively drug-resistant; MS/MS, tandem mass spectrometry; P, patient; PCT, procalcitonin; SCr, serum creatinine; UO, urine output; UV, ultra-violet spectroscopy

References

39. Fournier A, Eggimann P, Pantet O et al. Impact of real-time therapeutic drug monitoring on the prescription of antibiotics in burn patients requiring admission to the intensive care unit. *Antimicrobial Agents and Chemotherapy* 2018; **62**.
40. De Waele JJ, Carrette S, Carlier M et al. Therapeutic drug monitoring-based dose optimisation of piperacillin and meropenem: a randomised controlled trial. *Intensive Care Med* 2014; **40**: 380-7.
43. Cies JF, Moore WS, Enache A et al. beta-lactam therapeutic drug management in the PICU. *Critical Care Medicine* 2018; **46**: 272-9.
44. Machado AS, Oliveira MS, Sanches C et al. Clinical Outcome and Antimicrobial Therapeutic Drug Monitoring for the Treatment of Infections in Acute Burn Patients. *Clinical Therapeutics* 2017; **39**: 1649-57.e3.
45. Economou CJP, Wong G, McWhinney B et al. Impact of beta-lactam antibiotic therapeutic drug monitoring on dose adjustments in critically ill patients undergoing continuous renal replacement therapy. *International Journal of Antimicrobial Agents* 2017; **49**: 589-94.
46. Fournier A, Eggimann P, Pagani JL et al. Impact of the introduction of real-time therapeutic drug monitoring on empirical doses of carbapenems in critically ill burn patients. *Burns* 2015; **41**: 956-68.
47. McDonald C, Cotta MO, Little PJ et al. Is high-dose beta-lactam therapy associated with excessive drug toxicity in critically ill patients? *Minerva Anestesiologica* 2016; **82**: 957-65.
48. Pea F, Della Siega P, Cojutti P et al. Might real-time pharmacokinetic/pharmacodynamic optimisation of high-dose continuous-infusion meropenem improve clinical cure in infections caused by KPC-producing Klebsiella pneumoniae? *International Journal of Antimicrobial Agents* 2017; **49**: 255-8.
49. Cojutti P, Maximova N, Pea F. Pharmacokinetics and pharmacodynamics of continuous-infusion meropenem in pediatric hematopoietic stem cell transplant patients. *Antimicrobial Agents & Chemotherapy* 2015; **59**: 5535-41.
50. Cojutti PG, Lazzarotto D, Candoni A et al. Real-time TDM-based optimization of continuous-infusion meropenem for improving treatment outcome of febrile neutropenia in oncohaematological patients: results from a prospective, monocentric, interventional study. *Journal of Antimicrobial Chemotherapy* 2020; **75**: 3029-37.
51. Patel BM, Paratz J, See NC et al. Therapeutic drug monitoring of beta-lactam antibiotics in burns patients--a one-year prospective study. *Ther Drug Monit* 2012; **34**: 160-4.
52. Wong G, Briscoe S, McWhinney B et al. Therapeutic drug monitoring of beta-lactam antibiotics in the critically ill: direct measurement of unbound drug concentrations to achieve appropriate drug exposures. *Journal of Antimicrobial Chemotherapy* 2018; **73**: 3087-94.
53. Roberts JA, Ulldemolins M, Roberts MS et al. Therapeutic drug monitoring of beta-lactams in critically ill patients: proof of concept. *Int J Antimicrob Agents* 2010; **36**: 332-9.
54. Bricheux A, Lenggenhager L, Hughes S et al. Therapeutic drug monitoring of imipenem and the incidence of toxicity and failure in hospitalized patients: a retrospective cohort study. *Clinical Microbiology & Infection* 2019; **25**: 383.e1-.e4.
55. Schoenenberger-Arnaiz JA, Ahmad-Diaz F, Miralbes-Torner M et al. Usefulness of therapeutic drug monitoring of piperacillin and meropenem in routine

clinical practice: A prospective cohort study in critically ill patients. *European Journal of Hospital Pharmacy* 2019; **27**: e30-e5.
56. Gatti M, Cojutti PG, Pascale R et al. Assessment of a PK/PD Target of Continuous Infusion Beta-Lactams Useful for Preventing Microbiological Failure and/or Resistance Development in Critically Ill Patients Affected by Documented Gram-Negative Infections. *Antibiotics-Basel* 2021; **10**: 12.
58. Aldaz A, Idoate Grijalba AI, Ortega A et al. Effectiveness of Pharmacokinetic/Pharmacodynamic-Guided Meropenem Treatment in Critically Ill Patients: A Comparative Cohort Study. *Therapeutic drug monitoring* 2021; **43**: 256-63.
57. Al-Shaer MH, Rubido E, Cherabuddi K et al. Early therapeutic monitoring of beta-lactams and associated therapy outcomes in critically ill patients. *Journal of Antimicrobial Chemotherapy* 2020; **75(12)**: 3644-51.
59. Scharf C, Liebchen U, Paal M et al. The higher the better? Defining the optimal beta-lactam target for critically ill patients to reach infection resolution and improve outcome. *Journal of intensive care* 2020; **8**: 86.
61. Gunasekaran K, Amladi A, Mathew S et al. A case of septicaemic melioidosis: Utility of therapeutic drug monitoring and high-dose meropenem in successful management. *Indian Journal of Medical Microbiology* 2018; **36**: 597-9.
62. Lonsdale DO, Udy AA, Roberts JA et al. Antibacterial therapeutic drug monitoring in cerebrospinal fluid: Difficulty in achieving adequate drug concentrations ; Case report. *Journal of Neurosurgery* 2013; **118**: 297-301.
63. Udy AA, Putt MT, Shanmugathasan S et al. Augmented renal clearance in the Intensive Care Unit: An illustrative case series. *International Journal of Antimicrobial Agents* 2010; **35**: 606-8.
64. Hayashi Y, Lipman J, Udy AA et al. beta-Lactam therapeutic drug monitoring in the critically ill: Optimising drug exposure in patients with fluctuating renal function and hypoalbuminaemia. *International Journal of Antimicrobial Agents* 2013; **41**: 162-6.
65. Wu YE, Xu HY, Shi HY et al. Carbapenem-Resistant Enterobacteriaceae Bloodstream Infection Treated Successfully With High-Dose Meropenem in a Preterm Neonate. *Front Pharmacol* 2020; **11**: 4.
66. Oda K, Kamohara H, Katanoda T et al. Continuous high-dose infusion of doripenem in a pneumonia patient infected by carbapenem-resistant Pseudomonas aeruginosa: a case report. *Journal of Pharmaceutical Health Care & Sciences* 2019; **5**: 15.
67. Troger U, Drust A, Martens-Lobenhoffer J et al. Decreased meropenem levels in Intensive Care Unit patients with augmented renal clearance: benefit of therapeutic drug monitoring. *International Journal of Antimicrobial Agents* 2012; **40**: 370-2.
68. Cotta MO, Gowen B, Truloff N et al. Even high-dose extended infusions may not yield desired concentrations of beta-lactams: the value of therapeutic drug monitoring. *Infectious Diseases* 2015; **47**: 739-42.
69. Cojutti PG, Barbarino C, De Monte A et al. Higher than standard meropenem and linezolid dosages needed for appropriate treatment of an intracerebral hemorrhage patient with augmented renal clearance. *European Journal of Clinical Pharmacology* 2018; **74**: 1091-2.
70. Afaneh CI, Ho VP, McWhorter P et al. Minor fluctuations in renal function may alter therapeutic drug concentrations substantially during high-dose, continuous-

infusion beta-lactam therapy for multi-drug-resistant Gram-negative bacilli. *Surgical Infections* 2012; **13**: 415-7.
71. Taccone FS, Cotton F, Roisin S et al. Optimal meropenem concentrations to treat multidrug-resistant Pseudomonas aeruginosa septic shock. *Antimicrobial Agents & Chemotherapy* 2012; **56**: 2129-31.
72. Pea F, Cojutti P, Sbrojavacca R et al. TDM-guided therapy with daptomycin and meropenem in a morbidly obese, critically ill patient. *Annals of Pharmacotherapy* 2011; **45**: e37.
73. Stewart A, Graves B, Hajkowicz K et al. The Use of Therapeutic Drug Monitoring to Optimize Treatment of Carbapenem-Resistant Enterobacter Osteomyelitis. *Microbial Drug Resistance-Mechanisms Epidemiology & Disease* 2015; **21**: 631-5.
74. Cies JJ, Moore WS, 2nd, Conley SB et al. Therapeutic Drug Monitoring of Continuous Infusion Doripenem in a Pediatric Patient on Continuous Renal Replacement Therapy. *The Journal of Pediatric Pharmacology & Therapeutics* 2017; **22**: 69-73.
75. De Keukeleire S, Borrey D, Decaluwe W et al. Therapeutic Drug Monitoring of Meropenem in Neonate with Necrotizing Enterocolitis: A Challenge. *Case Reports Infectious Diseases* 2016; **2016**: 6207487.
76. Liebchen U, Paal M, Jung J et al. Therapeutic drug monitoring-guided high dose meropenem therapy of a multidrug resistant Acinetobacter baumannii - A case report. *Respiratory Medicine Case Reports* 2020; **29**: 100966.
77. Goutelle S, Conrad A, Pouderoux C et al. Pharmacokinetic/Pharmacodynamic Dosage Individualization of Suppressive Beta-Lactam Therapy Administered by Subcutaneous Route in Patients With Prosthetic Joint Infection. *Frontiers in medicine* 2021; **8**: 583086.
78. Legg A, Halford M, McCarthy K. Plasma concentrations resulting from continuous infusion of meropenem in a community-based outpatient program: A case series. *American journal of health-system pharmacy : AJHP : official journal of the American Society of Health-System Pharmacists* 2020; **77**: 2074-80.
79. Cojutti PG, Fornaro G, Gatti M et al. Successful Treatment of Bacteremia and Ventilator-Associated Pneumonia Caused by KPC/OXA-48-like Klebsiella pneumoniae Co-Producer with a Continuous Infusion of High-Dose Meropenem Plus Fosfomycin Guided by Real-Time Therapeutic Drug Monitoring. *Infectious Disease Reports* 2022; **14**: 88-92.
